# Supplementary material for: The Role of Intention, Behavioral Regulation, and Physical Activity Behavior in the Prediction of Physical Activity Identity across Time
Source: Behav Sci (Basel). 2024 Oct 1;14(10):886. doi: 10.3390/bs14100886 (PMC11505567; doi:10.3390/bs14100886)
Supplement: Supplementary file 1 [file behavsci-14-00886-s001.zip › behavsci-3108778-supplementary.pdf]

Supplementary Tables

Table S1. Items from multi-item scales.

Construct	Item
Integrated regulation	I exercise because it is consistent with my life goals
	I consider exercise part of my identity
	I consider exercise a fundamental part of who I am
	I consider exercise consistent with my values
Behavioral regulation	I kept track of my physical activity in a diary or log over the last 2 weeks
	I made regular plans concerning “when”, “where”, “how”, and “what” kind of physical activity I did the last 2 weeks
	I made plans regarding what to do if something interfered with my engaging in physical activity last 2 weeks
	I reserved time in my daily schedule for regular leisure-time physical activity

Table S2. Bivariate correlations, descriptive statistics, and reliability statistics for study variables.

	t1.ID	t1.Int	t1.BH	t1.PA	t2.ID	t2.Int	t2.BH	t2.PA	t3.ID	t3.Int	t3.BH	t3.PA	t4.ID	t4.Int	t4.BH	t4.PA
t1.ID	--															
t1.Int	0.29*	--														
t1.BH	0.59***	0.18	--													
t1.PA	0.26*	0.22	0.38**	--												
t2.ID	0.67***	0.16	0.65***	0.28*	--											
t2.Int	0.19	0.40**	-0.03	0.11	0.01	--										
t2.BH	0.36*	0.17	0.38**	0.33*	0.49***	0.32*	--									
t2.PA	0.44**	0.13	0.25	0.51***	0.37**	0.28	0.58***	--								
t3.ID	0.72***	0.39*	0.63***	0.35*	0.77***	0.06	0.58***	0.47**	--							
t3.Int	0.47**	0.51***	0.24	0.04	0.52**	0.15	0.25	0.42*	0.39*	--						
t3.BH	0.33*	0.24	0.28	0.01	0.37*	0.08	0.85***	0.53**	0.59***	0.43**	--					
t3.PA	0.17	0.13	0.40*	0.39*	0.41*	-0.09	0.51**	0.62***	0.39*	0.17	0.49**	--				
t4.ID	0.61***	0.29	0.43**	0.27	0.86***	-0.16	0.34*	0.31	0.80***	0.42*	0.44*	0.34	--			
t4.Int	0.22	0.13	0.00	-0.10	0.39*	0.14	0.26	0.29	0.18	0.35	0.34	0.22	0.41*	--		
t4.BH	0.38*	-0.06	0.00	-0.08	0.40*	-0.23	0.35*	0.26	0.38*	0.32	0.49**	0.08	0.35*	0.33*	--	
t4.PA	-0.04	-0.16	-0.13	0.21	0.03	-0.19	0.21	0.26	-0.03	0.09	0.12	0.43*	-0.04	0.42**	0.29	--
M (SD)	2.65 (0.83)	1.65 (1.23)	0.23 (1.04)	107.20 (96.27)	2.51 (1.01)	1.42 (1.09)	-0.38 (1.17)	124.48 (93.02)	2.74 (0.91)	1.53 (1.18)	-0.46 (1.26)	115.25 (95.33)	2.93 (0.77)	2.05 (1.06)	0.46 (1.30)	147.50 (77.20)
α	0.82		0.81		0.90		0.78		0.90		0.77		0.84		0.71	

Note: t1 = baseline, t2 = week 3, t3 = week 6, t4 = week 9. ID = physical activity identity, Int = Intention, BH = behavioral regulation, PA = moderate-to-vigorous physical activity. $p < 0.05$*, $p < 0.01$**, $p < 0.001$***. M = mean, SD = standard deviation. α = Cronbach's alpha.

Table S3. Time models.

Variable	Model 0 *b* (SE)	*p*-value	Model 1 *b* (SE)	*p*-value	Model 2 *b* (SE)	*p*-value
Intercept	2.57 (0.09)	<0.0001	2.64 (0.11)	<0.0001	2.63 (0.11)	<0.0001
Time	0.10 (0.05)	0.08	-0.15 (0.19)	0.43	0.07 (0.04)	0.13
Time^2			0.08 (0.06)	0.18		
ICC					0.74	
Model fit						
AIC	520.47		520.62		436.38	
BIC	530.37		533.81		449.53	

Note: Model 2 included a random intercept. SE = standard error. ICC = intraclass correlation coefficient.

Table S4. Intention models.

**Variable**	**Model 0**<br>***b* (SE)**	***p*-value**	**Model 1**<br>***b* (SE)**	***p*-value**	**Model 2**<br>***b* (SE)**	***p*-value**
Intercept	2.25 (0.13)	<0.0001	2.54 (0.13)	<0.0001	2.55 (0.15)	<0.0001
Time	0.08 (0.06)	0.17	0.06 (0.05)	0.21	0.05 (0.08)	0.56
Intention	0.20 (0.05)	<0.001	0.05 (0.05)	0.27	0.04 (0.06)	0.48
Time*Intention					0.01 (0.04)	0.87
ICC			0.71			0.71
Model fit						
AIC	477.71		424.04		430.71	
BIC	490.59		440.06		449.91	

Note: Models 1 and 2 included a random intercept. SE = standard error. ICC = intraclass correlation coefficient.

Table S5. Behavioural regulation models.

**Variable**	**Model 0**<br>***b* (SE)**	***p*-value**	**Model 1**<br>***b* (SE)**	***p*-value**	**Model 2**<br>***b* (SE)**	***p*-value**
Intercept	2.57 (0.08)	<0.0001	2.59 (0.09)	<0.0001	2.58 (0.09)	<0.0001
Time	0.09 (0.05)	0.08	0.07 (0.04)	0.12	0.08 (0.05)	0.10
Behavioural regulation	0.38 (0.05)	<0.0001	0.26 (0.04)	<0.0001	0.29 (0.07)	<0.0001
Time*Behavioural regulation					-0.02 (0.04)	0.59
ICC			0.64		0.64	
Model fit						
AIC	428.69		394.08		400.62	
BIC	441.55		410.07		419.78	

Note: Models 1 and 2 included a random intercept. SE = standard error. ICC = intraclass correlation coefficient.

Table S6. MVPA models.

**Variable**	**Model 0**<br>***b* (SE)**	***p*-value**	**Model 1**<br>***b* (SE)**	***p*-value**	**Model 2**<br>***b* (SE)**	***p*-value**
Intercept	2.26 (0.12)	<0.0001	2.57 (0.13)	<0.0001	2.57 (0.14)	<0.0001
Time	0.08 (0.06)	0.16	0.06 (0.05)	0.22	0.05 (0.08)	0.51
MVPA	0.003 (0.0007)	<0.001	0.001 (0.0006)	0.42	0.0005 (0.0008)	0.56
Time*MVPA					0.00004 (0.0005)	0.94
ICC			0.70		0.70	
Model fit						
AIC	476.06		432.23		447.52	
BIC	488.92		448.22		466.68	

Note: Models 1 and 2 included a random intercept. SE = standard error. ICC = intraclass correlation coefficient.

Table S7. Exploratory final model controlling for demographic variables.

Variable	Beta coefficient	Standard error	*p*-value
Intercept	2.55	0.37	<0.0001
Time	0.09	0.05	0.08
Intention	-0.03	0.05	0.53
Behavioral regulation	0.23	0.05	<0.0001
MVPA	-0.0002	0.0007	0.82
Age	0.003	0.01	0.72
Gender	-0.11	0.20	0.59
Minority status	-0.26	0.26	0.31
	ICC		
	0.66		

Note: ICC = intraclass correlation coefficient.
